# Partially dissecting the steady-state electron fluxes in Photosystem I in wild-type and *pgr5* and *ndh* mutants of *Arabidopsis*

**DOI:** 10.3389/fpls.2015.00758

**Published:** 2015-09-17

**Authors:** Jiancun Kou, Shunichi Takahashi, Da-Yong Fan, Murray R. Badger, Wah S. Chow

**Affiliations:** ^1^College of Animal Science and Technology, Northwest A&F UniversityYangling, China; ^2^Division of Plant Sciences, Research School of Biology, The Australian National UniversityCanberra, ACT, Australia; ^3^State Key Laboratory of Vegetation and Environmental Change, Institute of Botany, The Chinese Academy of SciencesBeijing, China

**Keywords:** antimycin A, *Arabidopsis*, cyclic electron flow, *ndh* mutant, P700, *pgr5* mutant, photosystem I

## Abstract

Cyclic electron flux (CEF) around Photosystem I (PS I) is difficult to quantify. We obtained the linear electron flux (LEF_O2_) through both photosystems and the total electron flux through PS I (ETR1) in *Arabidopsis* in CO_2_-enriched air. ΔFlux = ETR1 – LEF_O2_ is an upper estimate of CEF, which consists of two components, an antimycin A-sensitive, PGR5 (proton gradient regulation 5 protein)-dependent component and an insensitive component facilitated by a chloroplastic nicotinamide adenine dinucleotide dehydrogenase-like complex (NDH). Using wild type as well as *pgr5* and *ndh* mutants, we observed that (1) 40% of the absorbed light was partitioned to PS I; (2) at high irradiance a substantial antimycin A-sensitive CEF occurred in the wild type and the *ndh* mutant; (3) at low irradiance a sizable antimycin A-sensitive CEF occurred in the wild type but not in the *ndh* mutant, suggesting an enhancing effect of NDH in low light; and (4) in the *pgr5* mutant, and the wild type and *ndh* mutant treated with antimycin A, a residual ΔFlux existed at high irradiance, attributable to charge recombination and/or pseudo-cyclic electron flow. Therefore, in low-light-acclimated plants exposed to high light, ΔFlux has contributions from various paths of electron flow through PS I.

## Introduction

Arnon et al. (1955) demonstrated photophosphorylation via a (CEF) around Photosystem I (PS I) by illuminating isolated thylakoids in the presence of vitamin K. Supply of ATP is one of the factors that limit photosynthesis, such that increasing cyclic photophosphorylation helps to increase photosynthetic rate (Shen, 1990). Since CEF is essential for efficient photosynthesis (Munekage et al., 2004) and for photoprotection (Munekage et al., 2002; Takahashi et al., 2009; Huang et al., 2011), there have been sustained efforts to elucidate the mechanisms and roles of this cyclic electron flow (for reviews, see Bendall and Manasse, 1995; Allen, 2003; Bukhov and Carpentier, 2004; Johnson, 2005; Joliot and Joliot, 2006; Shikanai, 2007, 2014; Alric, 2010; Miyake, 2010; Kramer and Evans, 2011; Leister and Shikanai, 2013; Johnson et al., 2014).

In flowering plants, cyclic electron flow consists of two pathways: one dependent on a PGR5–PGRL1 protein interaction (i.e., interaction between the proton gradient regulation 5 protein and the PGR5-like protein 1) and the other on a chloroplastic nicotinamide adenine dinucleotide dehydrogenase-like complex (NDH) complex. It is likely that the PGR5–PGRL1-dependent pathway, inhibitable by antimycin A, corresponds to the cyclic photophosphorylation discovered by Arnon et al. (1955). Indeed, Hertle et al. (2013) showed that PGRL1 accepts electron from reduced ferredoxin in a PGR5-dependent manner and reduces quinones in an antimycin A-sensitive manner, proposing that PGRL1 is the elusive ferredoxin-plastquinone reductase. In the absence of PGR5, the chloroplast NDH-dependent pathway compensates for the loss of the important pathway to some extent (Munekage et al., 2004; Shikanai, 2014). For example, NDH may contribute to redox homeostasis in chloroplasts at low irradiance (Shikanai, 2014). Specifically, the NDH complex improves CEF by adjusting the redox level of electron carriers in low light (Martin et al., 2015).

Nevertheless, the involvement of these complexes in cyclic electron flow is complex. Even the antimycin A-sensitive pathway itself is still controversial (Leister and Shikanai, 2013). For example, there was some doubt as to whether the PGR5 protein is essential for cyclic electron flow at all (Nandha et al., 2007), though the technique for assaying CEF may be problematic (Leister and Shikanai, 2013). Certainly, understanding CEF has been hampered by the difficulty of quantifying CEF *in vivo* in physiological conditions due to the absence of a net product of cyclic electron flow. Methods for measuring/inferring CEF have all suffered from deficiencies. Kou et al. (2013) used a method that (a) estimates the total electron flux through PS I (ETR1) from the PS I photochemical yield, and the linear electron flux through both photosystems from the gross rate of oxygen evolution (LEF_O2_) under identical illumination, both being whole-tissue measurements; (b) uses white actinic light to simulate sunlight, since colored light alters CEF; (c) took note of the important finding that a proper determination of the photochemical yield of PS I for calculating the total electron flux through PS I requires strong far-red light immediately before and during the application of a saturating light pulse (Siebke et al., 1997); and (d) is non-intrusive. This method is not yet ideal, being applicable only to CO_2_-enriched air, but it yields a reasonable quantitative estimate of CEF in spinach leaf disks from glasshouse plants.

In this study, the same method was applied to leaf disks of *Arabidopsis* plants grown in low-light in a controlled-environment chamber. We used wild-type as well as *pgr5* and *ndh* mutants, in the absence or presence of antimycin A. The aim of this study was to attempt to semi-quantitatively dissect the electron fluxes that pass through PS I at varied irradiance.

## Materials and Methods

### Plant Growth

*Arabidopsis thaliana* wild-type (ecotype Columbia) and *pgr5* and *ndh* mutants were grown in a controlled-environment chamber at an irradiance of 100 μmol photons m^-2^ s^-1^ at 22°C, with a light/dark cycle of 10/14 h at 22°C. At 3–4 weeks from germination, fully expanded leaves were used for experiments.

### Vacuum Infiltration of Leaf Disks

When required, leaf disks (1.5 cm^2^) were immersed in water containing 0.2% dimethylsulfoxide or a 200 μM-solution of antimycin A with 0.2% dimethylsulfoxide carried over from the undiluted stock solution, vacuum-infiltrated using a water-driven pump for about 30 s, blotted with absorbent paper, and allowed to evaporate off the excess intercellular water in darkness for a total of 30 min before measurement.

### Linear Electron Flux Measured by O_2_ Evolution

O_2_ evolution was measured in a gas-phase oxygen electrode (Hansatech, King’s Lynn, UK) chamber, thermostated at 25°C, that accepted a multifurcated light guide with five arms, and contained 1% CO_2_ supplied by fabric matting moistened with 1 M NaHCO_3_/Na_2_CO_3_ (pH 9). White incandescent light from a projector halogen lamp filtered by a Calflex C heat-reflecting filter (Linos Photonics, Göttingen, Germany) and neutral-density filters was used to illuminate a leaf disk. O_2_ evolution was measured over several minutes until steady state. The post-illumination drift was subtracted algebraically from the steady-state net oxygen evolution rate, and the *gross* oxygen evolution rate so obtained was multiplied by four to give the linear electron flux, LEF_O2_. For calibration of the oxygen signals, 1 mL of air at 25°C (taken to contain 8.05 μmol O_2_) was injected into the gas-phase O_2_ electrode chamber.

### Measurement of Redox Kinetics of P700

Redox changes of P700, the special Chl dimer acting as the primary electron donor in PS I, were observed with a dual wavelength (820/870 nm) unit (ED-P700DW) attached to a PAM fluorometer (Walz, Effeltrich, Germany) in the reflectance mode (response time constant = 95 μs), as described by Kou et al. (2013). Lights and signals were transmitted through the multifurcated light guide inserted into the oxygen electrode. Before measurements, a leaf disk was brought to steady-state photosynthesis by illuminating it with white actinic light for about 10 min during which O_2_ evolution was measured. To retain steady state illumination for P700^+^ measurements, immediately after O_2_ measurement, each leaf disk was re-illuminated with the same actinic light for 9.016 s, using an electronic shutter controlled by one terminal of a pulse/delay generator (Model 565, Berkeley Nucleonics, USA). In this way, oxygen evolution and P700 redox kinetics were measured sequentially, both during steady-steady actinic illumination at a selected irradiance, under identical conditions in 1% CO_2_ and at 25°C. To improve the P700^+^ signal-to-noise ratio, the 9.016-s illumination was repeated nine times, with < 0.88 s dark time between repeats so as maintain steady state photosynthesis, as described by Kou et al. (2013).

Near the end of each 9.016-s illumination, data acquisition by a computer program was initiated by a trigger pulse from another terminal of the pulse/delay generator, followed (50 ms later) by the firing of an LED far-red pulse (∼800 μmol photons m^-2^ s^-1^) to oxidize the inter-system electron carriers with a duration of 100 ms during which was added a saturating pulse of white LED light (∼9000 μmol photons m^-2^ s^-1^, 10 ms duration) to maximally oxidize all the P700 that can be oxidized while the actinic light was on. On application of the two pulses, the P700^+^ signal height reached the level *P*_m_′ in the presence of actinic light. In a separate measurement afterward, continuous weak far-red light (∼50 μmol photons m^-2^ s^-1^) was applied to oxidize ∼85% of the P700; then a saturating xenon flash was given to oxidize the remaining P700, giving the maximum signal height (*P*_m_) in the presence of weak far-red light. The height of the P700^+^ signal in actinic light, before the application of far-red and white saturating pulses, is denoted as *a*, the increase in the P700^+^ signal height on application of the two pulses as *b*, and (*P_m_* - *P_m_*′) as *c* (see Klughammer and Schreiber, 2008). Thus, the photochemical yield of PS I, Y(I), was measured as described by Kou et al. (2013) by a slight modification of the method of Klughammer and Schreiber (2008) by adding a strong far-red pulse. Following Klughammer and Schreiber (2008), the photochemical yield of PS I is Y(I) = *b*/(*a* + *b* + *c*), averaged over closed PS I traps (containing P700^+^) and open PS I traps (containing P700). The non-photochemical yield due to donor-side limitation is Y(ND) = *a*/(*a* + *b* + *c*) and the non-photochemical yield due to acceptor-side limitation is Y(NA) = *c*/(*a* + *b* + *c*). Y(I) + Y(ND) + Y(NA) = 1

The total electron flux through PS I, ETR1, was calculated as

(1) $$ETR1\,\;=\;Y\left(I\right)\times I\times 0.85\times f_{I}\,\;$$

where *I* is the irradiance, 0.85 is the assumed absorptance of the leaf disk and *f*_I_ is the fraction of absorbed white light partitioned to PS I. An experimental estimation of *f*_I_ at low irradiance and in the presence of antimycin A is given in **Table 1**.

**Table 1:** Estimation of the fraction of absorbed light (*f*_I_) partitioned to PS I.

Leaf disks (+antimy. A)	Irradiance (μmol m^-2^ s^-1^)	Y(I)	LEF_O2_ (μmol e^-^ m^-2^ s^-1^)	*f*_I_
Wild type	135	0.746 ± 0.020	33.1 ± 1.5	0.39
	203	0.563 ± 0.029	41.0 ± 2.3	0.42
	265	0.488 ± 0.031	40.6 ± 0.8	0.37
				**Av = 0.39**
*pgr5*	135	0.569 ± 0.037	27.4 ± 1.4	0.42
	203	0.507 ± 0.022	35.5 ± 2.0	0.41
	265	0.475 ± 0.017	37.8 ± 1.8	0.35
				**Av = 0.39**
*ndh*	135	0.620 ± 0.060	28.8 ± 3.0	0.40
	203	0.506 ± 0.054	31.8 ± 2.5	0.36
	265	0.439 ± 0.020	34.9 ± 2.4	0.35
				**Av = 0.37**

*Y(I) is the photochemical yield of PS I, and LEF_O2_ is the linear electron flux determined by the gross rate of O_2_ evolution. Values are mean ± SE. (n = 4 leaf disks for the wild type, seven for pgr5, and three for the ndh mutant). The value of f_I_ is estimated from LEF_O2_ = Y(I) × I × 0.85 × f_I_, where I is irradiance, assuming that there is neither CEF nor charge recombination at low irradiance combined with infiltration with a solution of 200 μM antimycin A. Av = Average.*

## Results and Discussion

The approach taken in this study has been to obtain the steady-state electron flux through PS I (ETR1) via the P700^+^ signal and the linear electron flux through both photosystems by oxygen evolution (LEF_O2_), at 25°C, in broad-spectrum white incandescent light and CO_2_-enriched air. The total electron flux through PS I and the linear electron flux were compared under identical conditions, both being whole-tissue measurements.

### Estimation of the Fraction of Absorbed Light (*f*_I_) Partitioned to PS I

To estimate the total electron flux through PS I according to Equation 1, it is necessary to estimate *f*_I_, the fraction of absorbed light partitioned to PS I. To estimate *f*_I_, we consider some special cases in which (a) CEF, (b) direct charge recombination in the PS I reaction center and (c) pseudo-cyclic electron flow associated with the water–water cycle initiated by the Mehler reaction are likely to be small or negligible. We assume that these conditions are approximately met when both (1) the irradiance is low (below 265 μmol photons m^-2^ s^-1^) and (2) antimycin A is present. Under this assumption, we equate LEF_O2_ with ETR1 [= Y(I) × *I* × 0.85 × *f*_I_] to obtain *f*_I_. It is seen that at three low irradiances, *f*_I_ averaged to be slightly below 0.4, in the wild type as well as in the *pgr5* and *ndh* mutants (**Table 1**). For simplicity, we took *f*_I_ to be 0.4, which is close to the values obtained at the lowest irradiance. Further, we assume that the partitioning of absorbed energy did not change at higher irradiances. In spinach, this appeared to be the case: *f*_I_ was ∼0.48 at an irradiance of 980 μmol photons m^-2^ s^-1^ in the presence of antimycin A, and about 0.47 at an irradiance ≤ 352 μmol photons m^-2^ s^-1^ in the absence of antimycin A (Kou et al., 2013).

### Wild Type

**FIGURE 1:**
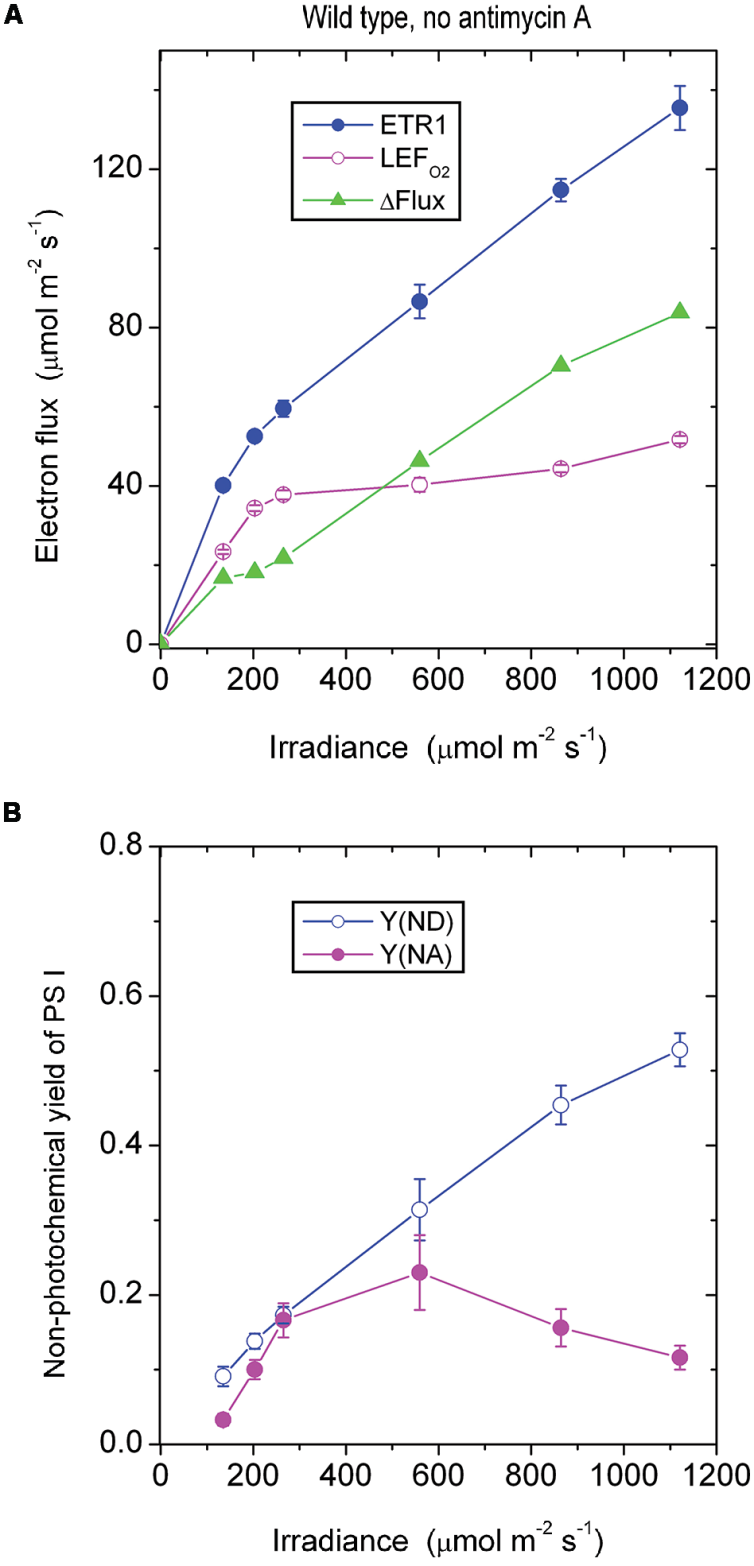
**(A)** Response of steady-state electron fluxes to irradiance of white light in the absence of antimycin A. ΔFlux = ETR1 - LEF_O2_. Each leaf disk was exposed to an irradiance that increased to the maximum using white halogen light filtered through neutral density filters. The leaf disk was maintained under each irradiance for about 10 min to reach steady-state photosynthesis. Oxygen evolution was first measured. Immediately afterward P700 kinetics were measured while maintaining steady-state photosynthesis. The temperature was 25°C. **(B)** The non-photochemical yield of PS I due to limitation on the donor side [Y(ND)] and the acceptor side [Y(NA)], measured simultaneously as ETR1 in **(A)**. Values are means ± SE. (*n* = 8 leaf disks).

In the *absence of antimycin A*, ETR1 (the total electron flux through PS I), calculated by assuming *f*_I_ = 0.4, did not show saturation even at the highest irradiance of white light used (**Figure 1A**). By contrast, LEF_O2_, assayed as the gross rate of O_2_ evolution multiplied by 4, almost peaked at about 250 μmol photons m^-2^ s^-1^, showing a slight increase at higher irradiances. The maximum LEF_O2_ reached about 50 μmol electrons m^-2^ s^-1^ (gross O_2_ evolution rate, about 12 μmol m^-2^ s^-1^). The difference between ETR1 and LEF_O2_ (=ΔFlux) increased approximately linearly with irradiance, even at low irradiance (**Figure 1A**). At the highest irradiance, ΔFlux exceeded LEF_O2_.

Wild type *Arabidopsis* grown in low light in a controlled chamber in this study behaved differently from spinach grown in a glasshouse. In spinach, LEF_O2_ did not reach a maximum until the irradiance was about 1500 μmol photons m^-2^ s^-1^; consequently, ETR1 and LEFO2 were approximately equal until the irradiance exceeded about 300 μmol photons m^-2^ s^-1^, above which ΔFlux increased approximately linearly (Kou et al., 2013). Therefore, whether a substantial ΔFlux exists at low irradiance appears to depend on how easily LEF_O2_ is saturated by light.

As the irradiance increased, P700 in wild-type *Arabidopsis* became more and more oxidized; consequently, fewer and fewer PS I complexes were able to perform charge separation because of the limitation due to P700^+^ on the donor side. Thus, the non-photochemical yield of PS I due to limitation on the donor side when P700 is oxidized, Y(ND), increased steadily with irradiance (**Figure 1B**). Interestingly, as the irradiance increased, the non-photochemical yield of PS I due to limitation on the acceptor side when the acceptors are reduced, Y(NA), first increased, and then declined above 559 μmol m^-2^ s^-1^. That is, at a high irradiance, electron carriers on the acceptor side seemed to be more oxidized. The enhanced oxidation of electron carriers on the acceptor side above 559 μmol m^-2^ s^-1^ probably resulted from a number of factors. One factor could be the hastening of downstream processes such as carbon assimilation, consistent with a further slight increase in LEF_O2_ above 559 μmol m^-2^ s^-1^ (**Figure 1A**). Other possible factors are discussed immediately below.

The steady increase of ΔFlux with irradiance could have at least three components. One is CEF that feeds electrons back to the PQ pool to be cycled through PS I. A second component is charge recombination: an electron on the acceptor side could recombine with P700^+^ on the donor side in a kind of short cycle, but this electron has had to be transferred through PS I in the first place, so it should be counted as part of the electron flux through PS I. A third component is the water–water cycle; although a complete water–water cycle leads to no net release or uptake of O_2_ and so does not affect the measurement of LEF_O2_ (Miyake, 2010), ETR1 should include (pseudo-cyclic) electron flow associated with the water-water cycle. Thus, ΔFlux = ETR1 - LEF_O2_ does include any pseudo-cyclic electron flux. As the irradiance increased, the observation that ΔFlux increased (**Figure 1A**) while Y(NA) decreased (**Figure 1B**) could come about if CEF, charge recombination and/or pseudo-cyclic electron flow increased, thereby alleviating the acceptor side limitation.

**FIGURE 2:**
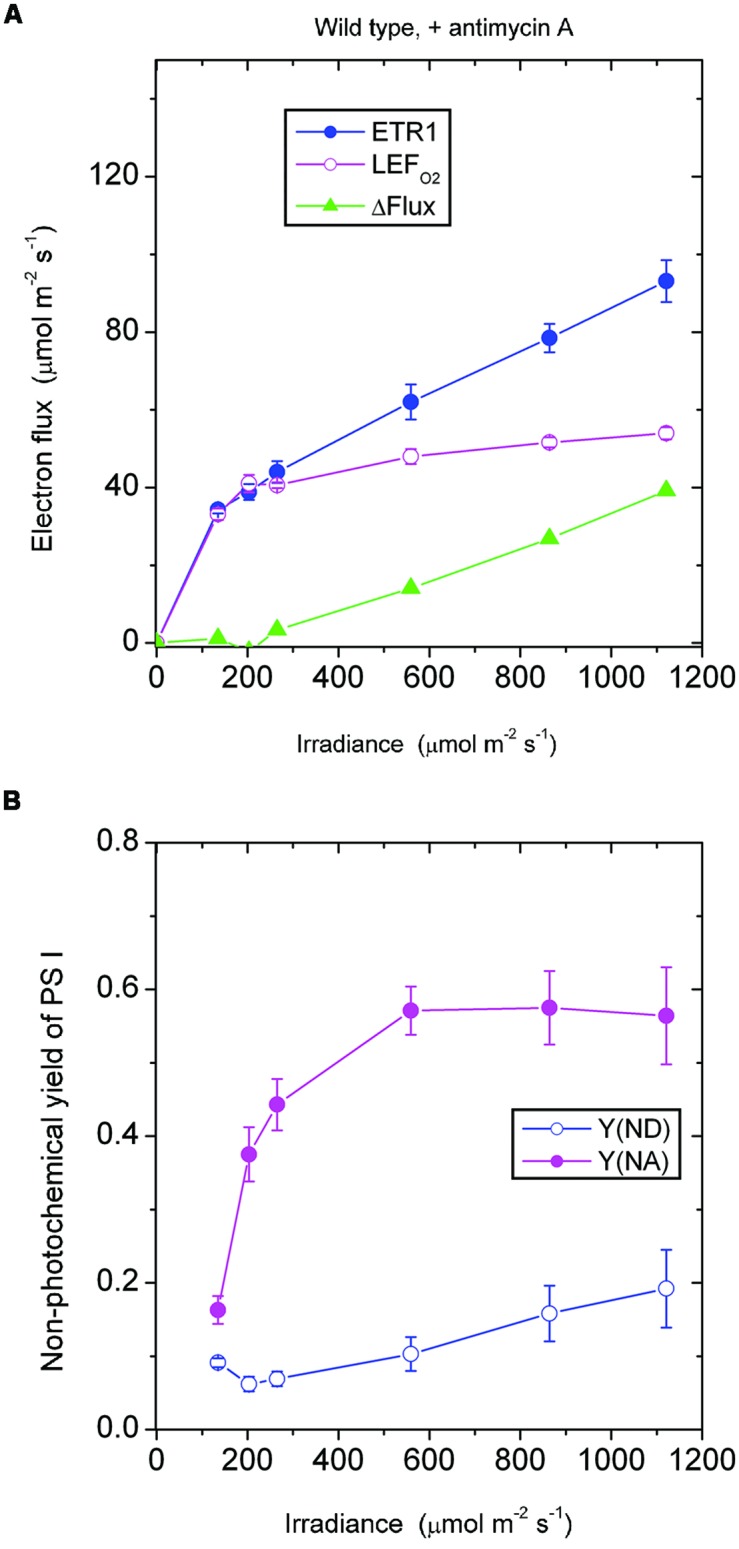
**Response of steady-state ETR1, LEF_O2_ and ΔFlux (A), and Y(ND) and Y(NA) (B) in wild-type leaf disks to irradiance in the presence of antimycin A.** Values are means ± SE. (*n* = leaf disks). Other conditions are as in **Figure 1**.

In the *presence of antimycin A*, the antimycin A-sensitive component of CEF should have been largely inhibited. Below 265 μmol photons m^-2^ s^-1^, ETR1 and LEF_O2_ were similar in magnitude (**Figure 2A**). Presumably below this irradiance, there was little or no pseudo-cyclic electron flux or charge recombination. Above this irradiance, however, ΔFlux increased steadily. Given that the acceptor side limitation was quite high at high irradiance, Y(NA) being ∼0.55, it is likely there was enhanced charge recombination and/or pseudo-cyclic electron flow due to a more reduced state of electron carriers on the acceptor side of PS I. Increased charge recombination might have been the more important of the two components of the electron flux in this situation: P700 was kept more reduced [lower Y(ND), **Figure 2B**] compared with the absence of antimycin A (**Figure 1B**), presumably by charge recombination, whereas pseudocyclic electron flow should have kept P700 more oxidized. Thus, it appears that inhibition of CEF by antimycin A resulted in a more reduced acceptor side, which in turn enhanced charge recombination and, potentially, also pseudo-cyclic electron flow at high irradiance.

It should be noted that low-light-grown plants such as those used in this study may be particularly prone to charge recombination when exposed to an irradiance 10-fold greater than their growth irradiance. They have neither the photosynthetic capacity to utilize the abundant photons nor the photoprotective capacity to dissipate excess excitation safely as heat. Consequently, charge recombination in PS I may be enhanced. In such low-light-grown plants, the low linear electron transport capacity would be readily saturated, accompanied by a low rate of carbon assimilation and any cyclic electron flux that occurred. Any excess electrons accumulating on the acceptor side of PS I would return to the donor side in a kind of a futile short circuit at the end of a linear chain and would constitute a component of ΔFlux not inhibited by antimycin A. This contrasts with glasshouse grown spinach which, in high light, has only a very small remaining ΔFlux in the presence of antimycin A (Kou et al., 2013).

### The *pgr5* Mutant

**FIGURE 3:**
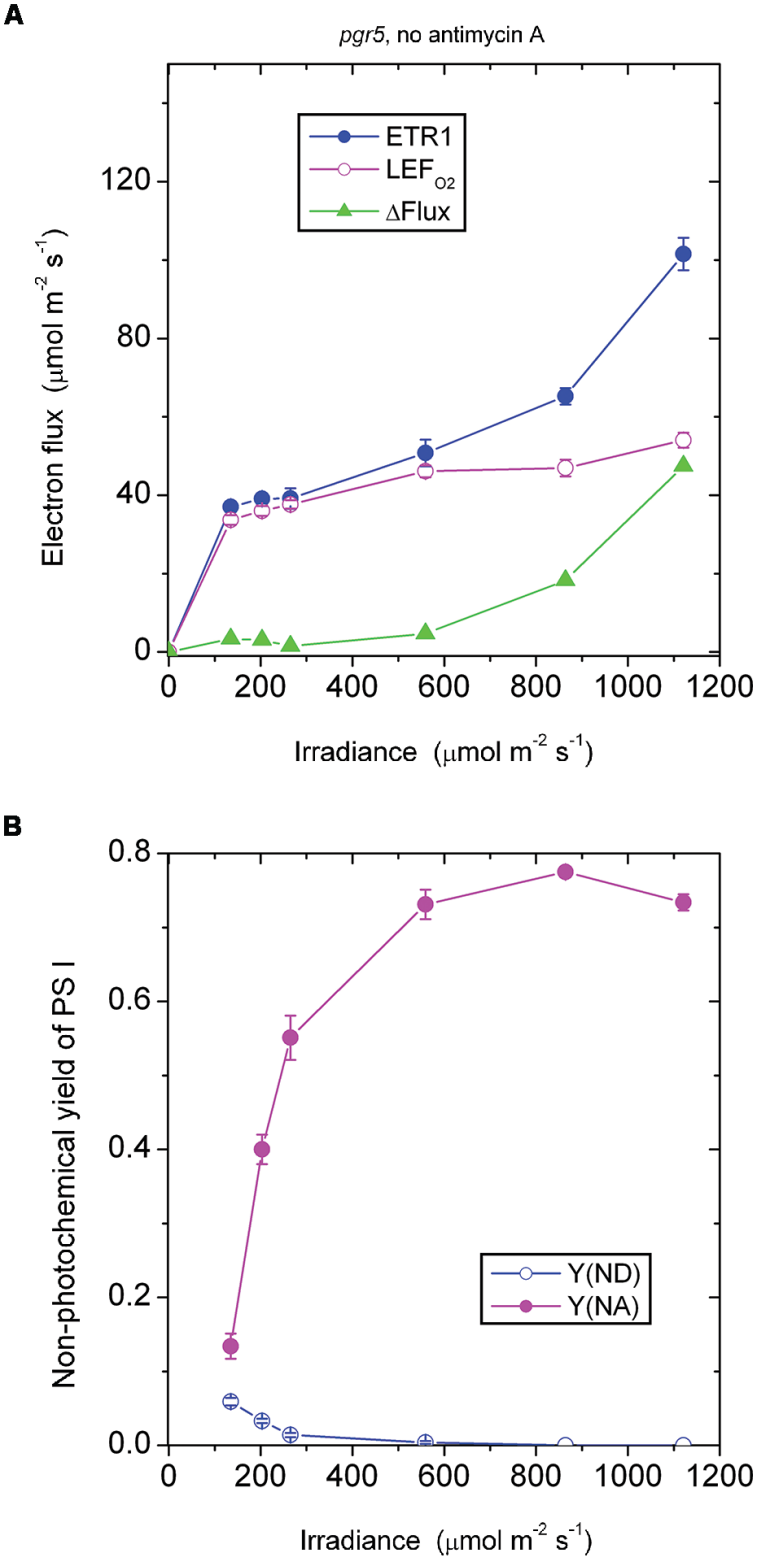
**Response of steady-state ETR1, LEF_O2_ and ΔFlux (A), and Y(ND) and Y(NA) (B) in leaf disks of the *pgr5* mutant to irradiance in the absence of antimycin A.** Values are means ± SE. (*n* = 16 leaf disks). Other conditions are as in **Figure 1**.

In the *absence of antimycin A*, the *pgr5* mutant showed a ΔFlux that only increased slowly with increase in irradiance (**Figure 3A**). For example, ΔFlux was only 4.7 μmol electrons m^-2^ s^-1^ at the irradiance 559 μmol photons m^-2^ s^-1^. This seems surprising, given that the acceptor side was highly reduced, Y(NA) being 0.73 (**Figure 3B**), which should have favored charge recombination and pseudo-cyclic electron flow. Probably, however, charge recombination could not occur because P700 was almost completely reduced and there was hardly any P700^+^ available for charge recombination. That is, the near-zero Y(ND) may be the reason for the very small ΔFlux at 559 above 559 μmol photons m^-2^ s^-1^. Further, this small ΔFlux, with a magnitude that was about 10% of LEF_O2_, also implies that pseudo-cyclic electron transfer was not very active at this irradiance despite the highly reduced state of the acceptor side.

Similarly, in the *pgr5* mutant at 1120 μmol photons m^-2^ s^-1^, the acceptor side was highly reduced and the donor side completely reduced (**Figure 3B**). At this irradiance, by contrast, ΔFlux was 47 μmol electrons m^-2^ s^-1^. At double the irradiance, many more excess electrons had to be discharged by either charge recombination or pseudo-cyclic electron transport in the form of increased ΔFlux (=47 μmol electrons m^-2^ s^-1^), but even so, Y(NA) and Y(ND) were each similar at 559 and 1120 μmol photons m^-2^ s^-1^ (**Figure 3B**).

**FIGURE 4:**
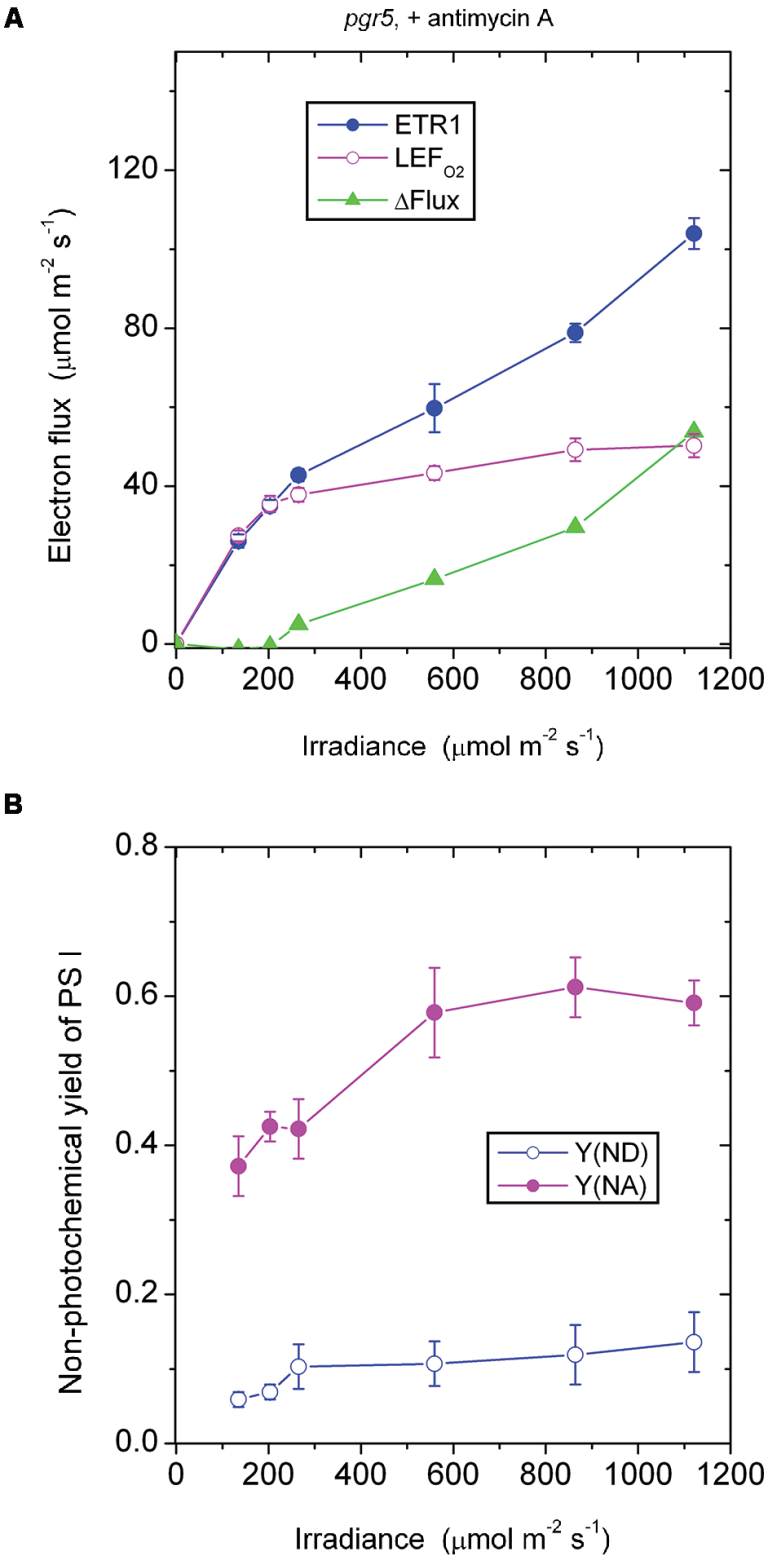
**Response of steady-state ETR1, LEF_O2_, and ΔFlux (A), and Y(ND) and Y(NA) (B) in leaf disks of the *pgr5* mutant to irradiance in the presence of antimycin A.** Values are means ± SE. (*n* = 7 leaf disks). Other conditions are as in **Figure 1**.

In the *presence of antimycin A*, the *pgr5* mutant (**Figure 4A**) behaved in a rather similar fashion as the wild type treated with antimycin A (**Figure 2A**) in response to increase in irradiance. In both cases, there was little or no ΔFlux below about 200 μmol photons m^-2^ s^-1^. This result implies that the ΔFlux ≈18 μmol electrons m^-2^ s^-1^ observed in the wild type in the absence of antimycin A (**Figure 1A**) at irradiance 135–203 μmol photons m^-2^ s^-1^ was due to antimycin A-sensitive CEF, the magnitude of which can be compared with the LEF_O2_ values: 23 μmol electrons m^-2^ s^-1^ at 135 μmol photons m^-2^ s^-1^, and 34 μmol electrons m^-2^ s^-1^ at 203 μmol photons m^-2^ s^-1^.

In both the *pgr5* mutant (**Figure 4A**) and the wild type (**Figure 2A**), in the presence of antimycin A and at irradiance 559 μmol photons m^-2^ s^-1^, there was a sizable ΔFlux (14–16 μmol electrons m^-2^ s^-1^. Since it appears that there was little or no pseudo-cyclic electron flux at this irradiance even when Y(NA) was much higher (**Figure 3A**), this sizable ΔFlux is attributed to charge recombination. At this irradiance of 559 μmol photons m^-2^ s^-1^, Y(ND) = 0.1, which was apparently sufficient to enable charge recombination to take place, thereby inducing a substantial ΔFlux in both the *pgr5* mutant and the wild type.

Comparing the absence (**Figure 3B**) or presence (**Figure 4B**) of antimycin A in the *pgr5* mutant, there was a clear difference at the lowest irradiance, 135 μmol photons m^-2^ s^-1^: Y(NA) was only 0.13 in the absence, but 0.37 in the presence of antimycin A. Perhaps the *pgr5* mutant, in the absence of antimycin A, was still capable of a small (antimycin A-sensitive) CEF which, at 135 μmol photons m^-2^ s^-1^, was able to maintain a largely oxidized state of electron carriers on the acceptor side. Nevertheless, when the irradiance increased further, this small CEF appeared to be abolished because of poor redox poising, Y(ND) approaching zero. At the same time, the acceptor side of PS I was mostly reduced, Y(NA) increasing to nearly 0.8 at the high irradiances.

Interestingly, when antimycin A was present in the *pgr5* mutant which hardly performs any CEF in the absence of antimycin A, Y(ND) was increased while Y(NA) was lowered after light saturation of linear electron flow, compared with the absence of the inhibitor. One possibility is that the binding of antimycin A somehow increased the Mehler reaction and/or plastid terminal oxidase (PTOX) reaction in the *pgr5* mutant; both reactions would increase Y(ND) and decrease Y(NA). PTOX is thought to act as a PQH_2_ water oxidoreductase (Johnson et al., 2014); if the superoxide formed in its reaction goes through in a complete water–water cycle, the linear electron flux measured by oxygen evolution is unaffected. Neither does the PTOX reaction affect the electron flux through PS I, so ΔFlux does not involve the PTOX reaction. However, electrons are shunted away by PTOX before reaching PS I, so that P700 is more oxidized, and the acceptor side less reduced.

The responses of Y(ND) and Y(NA) of the *pgr5* mutant (in which CEF is mostly inhibited even in the absence of antimycin A) to the treatment with antimycin A differed from the responses in the wild type and (see later) the *ndh* mutant. In both the wild type and the *ndh* mutant, antimycin A decreased Y(ND) but increased Y(NA). This can be rationalized in two ways. First, CEF was inhibited by antimycin A, so that electrons accumulated on the acceptor side, thereby increasing Y(NA). Second, the lower trans-thylakoid pH gradient on inhibition of CEF allowed electrons to be transferred from PQH2 more readily through to P700^+^, thereby decreasing Y(ND).

### The *ndh* Mutant

**FIGURE 5:**
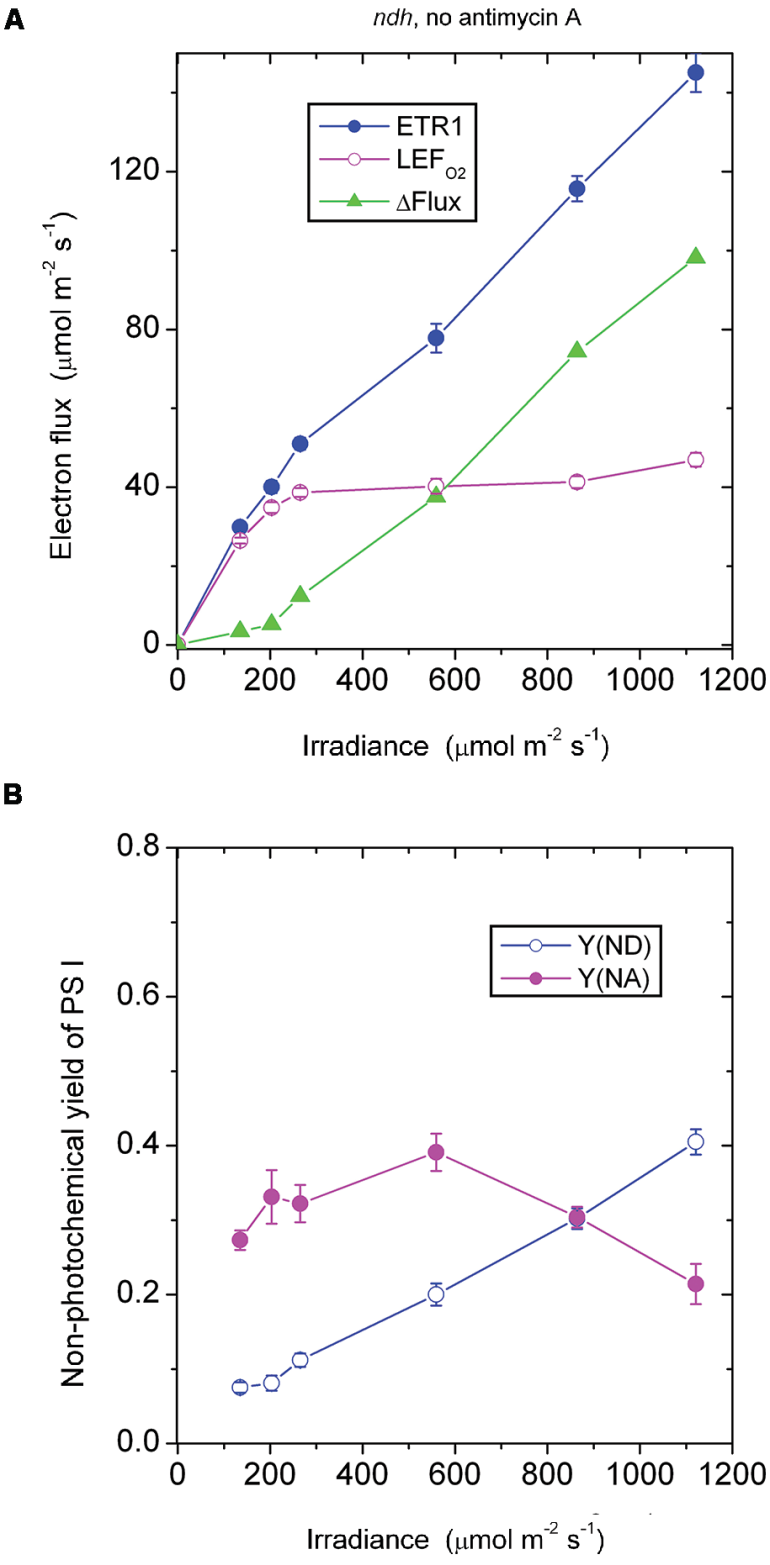
**Response of steady-state ETR1, LEF_O2_, and ΔFlux (A), and Y(ND) and Y(NA) (B) in leaf disks of the *ndh* mutant to irradiance in the absence of antimycin A.** Values are means ± SE. (*n* = 4 leaf disks). Other conditions are as in **Figure 1**.

In the *absence of antimycin A*, the *ndh* mutant should still be capable of generating the CEF that is sensitive to antimycin A. As expected, ΔFlux increased steadily when the irradiance was above 203 μmol photons m^-2^ s^-1^ (**Figure 5A**). This component of CEF maintained electron carriers on the acceptor side of PS I in a partially oxidized state, Y(NA) being between 0.2 and 0.4 (**Figure 5B**).

At an irradiance ≤ 203 μmol photons m^-2^ s^-1^, ΔFlux in the *ndh* mutant in the absence of antimycin A was low (**Figure 5A**), considerably lower than ΔFlux in the wild type in the absence of antimycin A (**Figure 1A**). It seems that NDH, which is present in the wild type, enhanced the antimycin A-sensitive component of CEF in the wild type, perhaps through redox regulation.

**FIGURE 6:**
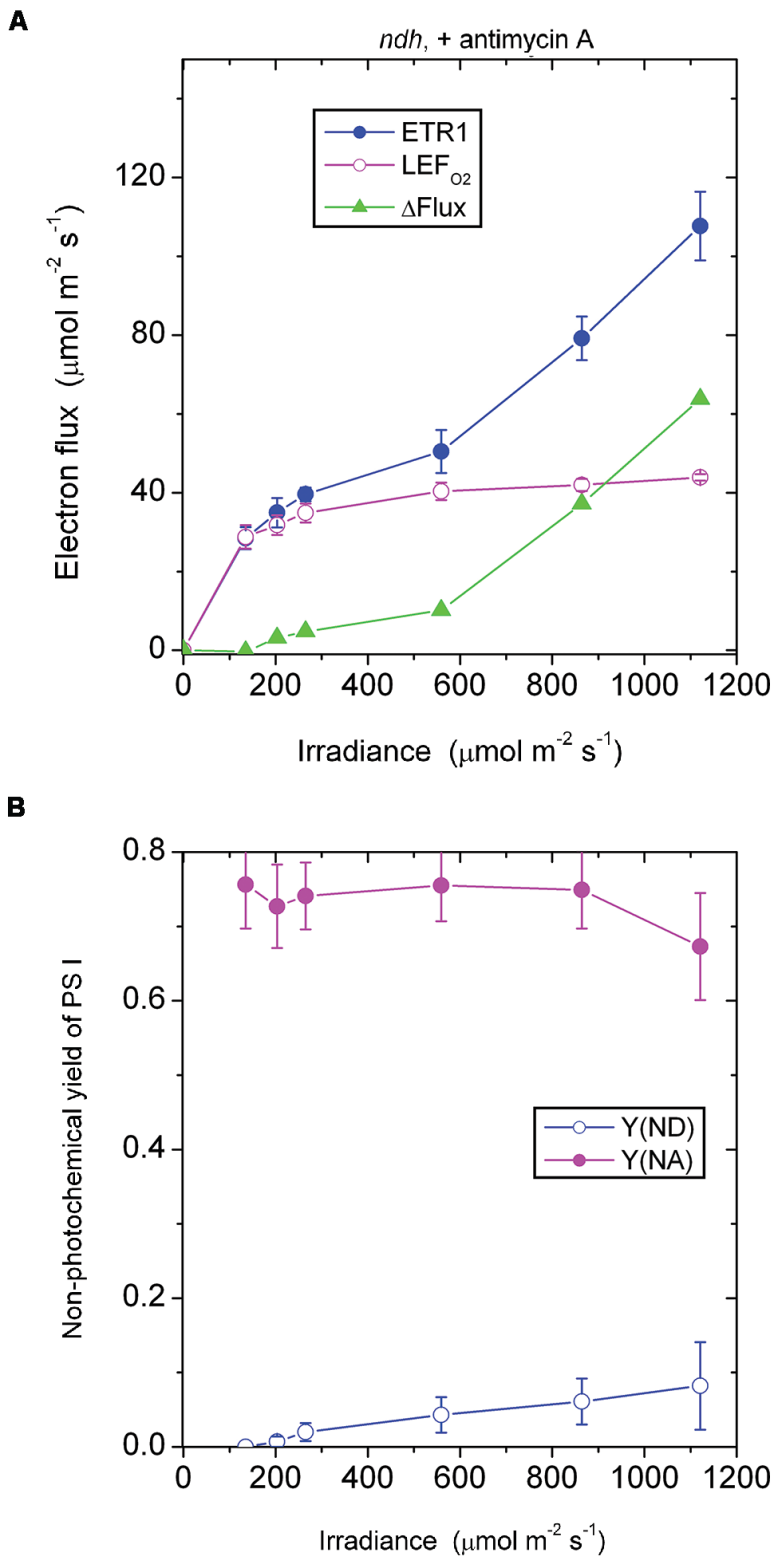
**Response of steady-state ETR1, LEF_O2_ and ΔFlux (A), and Y(ND) and Y(NA) (B) in leaf disks of the *ndh* mutant to irradiance in the presence of antimycin A.** Values are means ± SE. (*n* = 3 leaf disks). Other conditions are as in **Figure 1**.

In the *presence of antimycin A*, the *ndh* mutant showed a large acceptor side limitation, Y(NA) being 0.7–0.75 throughout the irradiance range. In particular, even when the irradiance was 135 μmol photons m^-2^ s^-1^, Y(NA) reached 0.76 (**Figure 6B**). By comparison, Y(NA) was only 0.37 in the *pgr5* mutant in the presence of antimycin A and at the same irradiance (**Figure 4B**). An obvious difference between (a) the *ndh* mutant in the presence of antimycin A and (b) the *pgr5* mutant in the presence of antimycin A is that the latter has NDH activity. It appears that when NDH-mediated CEF is permitted, over-reduction of the acceptor side is prevented. Thus, NDH may act as a safety valve when the stroma or, indeed, the acceptor side of PS I, is highly reduced; that is, NDH may contribute to redox homeostasis in chloroplasts at low irradiance (Shikanai, 2014).

### The Antimycin A-Sensitive Component of ETR1

**FIGURE 7:**
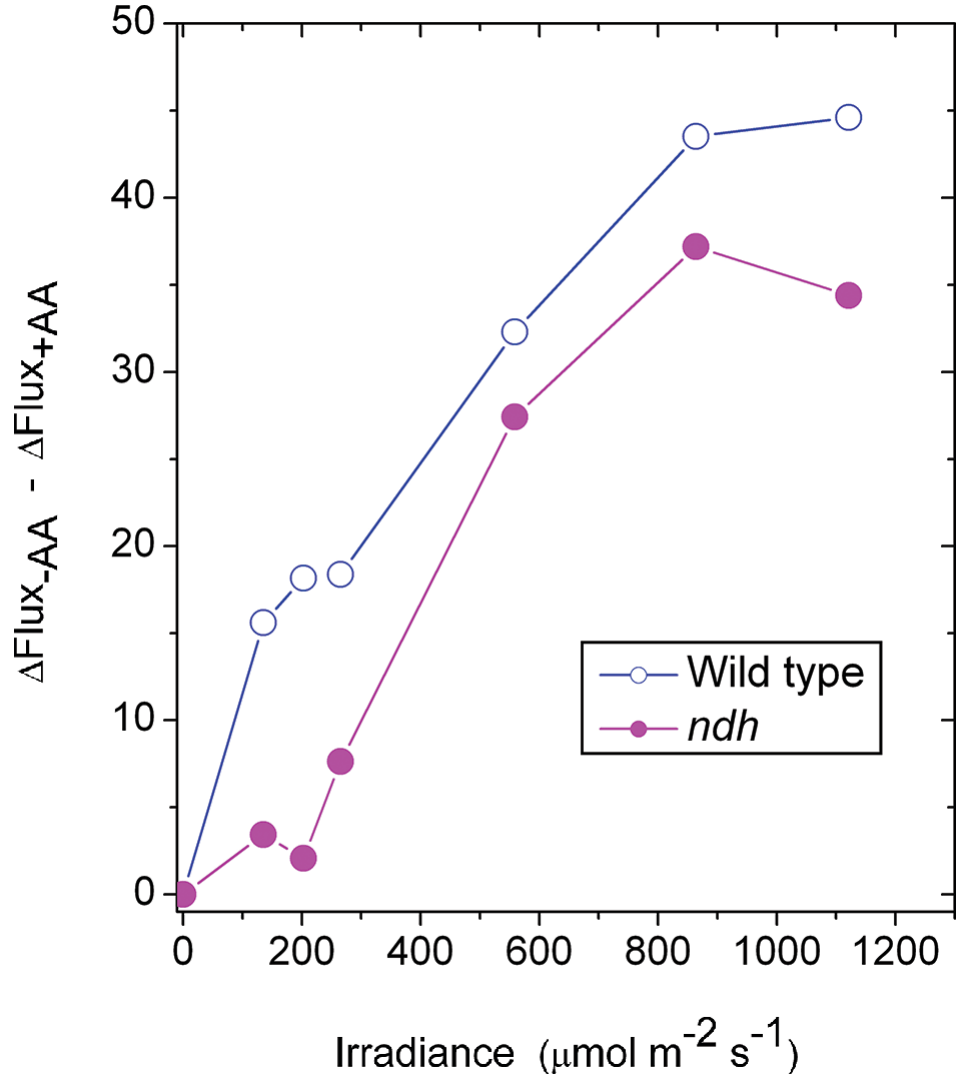
**The difference between ΔFlux in the absence of antimycin (-AA) and the presence of antimycin A (+AA), representing an underestimate of the component of CEF inhibitable by antimycin A, is plotted against irradiance.** The data are derived from **Figures 1A** and **2A** for the wild type and from **Figures 5A** and **6A** for the *ndh* mutant.

It may be instructive to estimate the magnitude of the antimycin A-sensitive component of ETR1. In the case of the wild type, the presence of antimycin A (+AA) gave a residual ΔFlux_+AA_ that is in general contributed by charge recombination, pseudo-cyclic electron flow and, possibly, the NDH-dependent cyclic flux (**Figure 2A**). Subtracting ΔFlux_+AA_ (**Figure 2A**) from ΔFlux_-AA_ (**Figure 1A**) gives an estimate of the antimycin A-sensitive component of ETR1. However, (ΔFlux_+AA_ - ΔFlux_-AA_) is an underestimate, since in the absence of antimycin A, the contributions from charge recombination and pseudo-cyclic electron flow could well have been smaller because of competition from CEF for electrons. Even when underestimated, the antimycin A-sensitive component of ETR1 was 15–18 μmol electrons m^-2^ s^-1^ at the three low irradiances, peaking at 45 μmol electrons m^-2^ s^-1^ at 864 μmol photons m^-2^ s^-1^ (**Figure 7**), where it is similar to the light-saturated LEF_O2_.

In the case of the *ndh* mutant, (ΔFlux_+AA_ - ΔFlux_-AA_) was only 4 μmol electrons m^-2^ s^-1^ when averaged over the lowest three irradiances, much lower than in the wild type. This suggests that the presence of NDH might have promoted the antimycin A-sensitive CEF in the wild type at low irradiance, perhaps via redox regulation (Martin et al., 2015). At the second highest irradiances, (ΔFlux_+AA_ - ΔFlux_-AA_) reached a peak value of 36 μmol electrons m^-2^ s^-1^. Thus, both the wild type and the *ndh* mutant were capable of antimycin A-sensitive CEF at rates comparable to LEF_O2_ at high irradiance. Further, large increases in antimycin A-sensitive CEF occurred at irradiances (∼200 μmol photons m^-2^ s^-1^) at which LEF_O2_ had been mostly light-saturated. Interestingly, the difference between (ΔFlux_+AA_ - ΔFlux_-AA_) of the wild type and that of the *ndh* mutant was 10 μmol electrons m^-2^ s^-1^ (approximately 25% of LEF_O2_), averaged over the entire irradiance range; this could be the cyclic electron flux contributed by NDH, indirectly (for example, by redox regulation that enhances antimycin A-sensitive CEF) or directly.

## Conclusion

A simple *non-intrusive* method is here presented that estimates *whole-tissue* ΔFlux under white light in CO_2_-enriched air. Application of the method to leaf disks of the wild type and the *pgr5* and *ndh* mutants of *Arabidopsis* yielded semi-quantitative estimates of (1) the antimycin A-sensitive CEF, (2) contribution of NDH to ETR1 and (3) possible contributions of charge recombination and/or pseudo-cyclic electron transport to the total electron flux through PS I, at varied irradiance. The electron fluxes through PS I in low-light-acclimated leaves exposed to unusually high light, unfortunately, have many components which cannot be easily dissected quantitatively.

## Conflict of Interest Statement

The Guest Associate Editor Wei Huang declares that, despite being affiliated with the same institution as the author Da-Yong Fan, the review process was handled objectively. The authors declare that the research was conducted in the absence of any commercial or financial relationships that could be construed as a potential conflict of interest.

## Abbreviations

ATP: adenosine triphosphate
CEF: cyclic electron flux around Photosystem I
Chl: chlorophyll
ETR1: the total electron transport rate through PS I
ΔFlux: the difference between ETR1 and LEF_O2_
*f*_I_: the fraction of absorbed light partitioned to PS I
LEF_O2_: the linear electron flux through PS II estimated by gross O_2_ evolution
NDH: nicotinamide adenine dinucleotide dehydrogenase-like complex
P700: special chlorophyll dimer acting as the primary electron donor in PS I
PAM: pulse amplitude modulation fluorometer
PGR5: proton gradient regulation 5 protein
PGRL1: PGR5-like protein 1
PS I: Photosystem I
PQH_2_: plastoquinonol
PTOX: plastid terminal oxidase
Y(I): photochemical yield of PS
Y(NA) and Y(ND): non-photochemical yield due to acceptor-side and donor-side limitation, respectively.
